# Moving beyond Risk Quotients: Advancing Ecological Risk Assessment to Reflect Better, More Robust and Relevant Methods

**DOI:** 10.3390/ecologies3020012

**Published:** 2022-05-27

**Authors:** Sandy Raimondo, Valery E. Forbes

**Affiliations:** 1 Gulf Ecosystem Measurement and Modeling Division, Office of Research and Development, United States Environmental Protection Agency, Gulf Breeze, FL 32561, USA; 2 Department of Ecology, Evolution and Behavior, University of Minnesota, St. Paul, MN 55108, USA

**Keywords:** risk management, mechanistic effect modeling, populations, chemical effects

## Abstract

Under standard guidance for conducting Ecological Risk Assessments (ERAs), the risks of chemical exposure to diverse organisms are most often based on deterministic point estimates evaluated against safety-factor-based levels of concern (LOCs). While the science and guidance for mechanistic effect models (e.g., demographic, population, and agent-based) have long been demonstrated to provide more ecologically relevant effect endpoints upon which risk can be evaluated, their application in ERAs has been limited, particularly in the US. This special issue highlights the state of the science in effect modeling for ERAs through demonstrated application of the recently published Population modeling Guidance, Use, Interpretation, and Development for ERA (Pop-GUIDE). We introduce this issue with a perspective on why it is critical to move past the current application of deterministic endpoints and LOCs. We demonstrate how the current, widely used approaches contain extensive uncertainty that could be reduced considerably by applying models that account for species life histories and other important endogenous and exogenous factors critical to species sustainability. We emphasize that it is long past time to incorporate better, more robust, and ecologically relevant effect models into ERAs, particularly for chronic risk determination. The papers in this special issue demonstrate how mechanistic models that follow Pop-GUIDE better inform ERAs compared to the current standard practice.

## 1. Introduction

Conducting an Ecological Risk Assessment (ERA) is a process by which measured or estimated environmental concentrations (EECs) of toxic chemicals are compared with the concentrations expected to cause adverse effects. The procedures used to conduct these assessments are governed by various regulatory bodies, and details may differ somewhat depending on regulatory jurisdiction [1]. Nevertheless, the objective of all assessments is to integrate chemical exposure and effects in a way that can inform environmental management and policy. The two overarching ways that the ERA process is used are to determine the safety of chemicals before they are put on the market (or periodically reviewed to allow them to remain in use) and to determine whether chemicals already in the environment may be causing harm to ecological systems. These are sometimes referred to as prospective and retrospective risk assessments, respectively.

In general, conducting an ERA is a tiered, iterative process that includes problem formulation (i.e., what chemicals and ecological receptors are considered), exposure analysis (i.e., use, fate, and distribution of chemical in the environment), effects analysis (i.e., adverse impacts), and risk characterization. The risk characterization of the assessment is the process in which risks are estimated and uncertainties are discussed. In practice, ERAs are associated with extensive uncertainties due to limited data on the concentrations of chemicals in the environment, the chemical effects measured on limited surrogate species, the translation of laboratory test conditions to the field, and the translation of effects on individuals to the impacts on populations [2,3]. The evaluation of most chemicals is initiated with a screening-level assessment that begins with conservative, worst-case assumptions about exposure and effects. If the initial assessment concludes a level of risk that is deemed unacceptable, the assumptions are refined for higher tiered assessments (which generally involve more time and effort) to make them more realistic. This tiered approach is designed to minimize effort involved in assessing clearly low-risk chemicals so that resources can be prioritized to provide robust assessments of chemicals likely to be higher risk [3,4] based on proposed or current uses.

The simplest, mostly widely applied method used to characterize chemical risks is performed by dividing a point estimate of exposure with a point estimate of effect to calculate a risk quotient (RQ), which is sometimes termed a deterministic hazard quotient [3,4]. The RQ is a dimensionless number that is then compared to a threshold value or level of concern (LOC) to determine a level of risk. Whether or not a level of risk is acceptable is based on the requirements of the regulatory statute under which the ERA is being conducted. As the next section will discuss in detail, there are inherent weaknesses in both RQs and LOCs, and the science has advanced sufficiently over the last 30 years such that other options are now available with which to gauge risk. Despite this, there has been a resistance by regulatory agencies to move beyond these measures of risk, even as the science continues to steadily advance [3]. For example, the USEPA’s general guidelines for conducting ERAs have not been updated since 1998 [4]. Although updated guidelines for specific applications (e.g., pollinators and endangered species) have been published more recently [5–7], even these updated guidelines continue to rely on RQs and LOCs to assess risk.

The purpose of this special issue of *Ecologies* on *Population Modeling for Ecological Risk Assessment and Management of Species* is to demonstrate recently published guidance for developing fit-for-purpose models that improve the characterization of chemical risks by integrating chemical exposure and effects in an ecologically meaningful way while incorporating appropriate levels of conservatism, realism, and precision to provide a robust basis for risk management and policy decisions. These models can replace RQs and LOCs with ecologically relevant probabilistic risk characterizations derived from integrating exposure model output with effects translated into impacts throughout species life cycles and resulting in population-level effects.

A main concern of decision makers in using population models as a basis for decision support has historically been a lack of guidance on model development, documentation, and evaluation. Without standardized approaches in these areas of model building and application, there can be multiple competing models that vary depending on how they are structured and the kinds of features that are included. Much progress has been made on these fronts over the years, starting with common standards for model documentation [8,9], followed by guidance on model evaluation [10]. More recent efforts have focused on developing guidance for explicitly linking population model complexity with the specific objectives of different types of regulatory risk assessments [11] and guidance to make the process of population model development more transparent and consistent [12]. Accolla et al. [13] provided a critical review of population models for use in the ERA process, with consideration of different model structures and the types of key features to include depending on the ERA context. The recently published Pop-GUIDE (Population modeling Guidance, Use, Interpretation, and Development for ERA) has taken these developments one step further in a continuing effort to demystify the process of population model development towards increasing the acceptance of such models as tools for risk characterization and decision support [14]. In what follows, we provide a comprehensive discussion of the shortcomings of current approaches used in the ERA process, with a focus on the US regulatory framework (Section 2). This section is emphasized in depth to demonstrate that deficiencies in the discrete components of exposure assessment, effects assessment, and risk characterization result in substantially flawed estimates of risk. We then provide a brief introduction to the kinds of population models useful for assessing the risks of chemicals on ecological receptors and the benefits of such models compared to current approaches (Section 3). This section is expanded on through the manuscripts included in this special issue of *Ecologies*. Finally, we provide some general take-home messages and guidelines for possible next steps that set the stage for advancing ERAs to reflect better, more robust and relevant methods (Section 4).

## 2. Background and Current Practices

Current US ecological risk characterization for pesticides relies heavily on RQs to estimate risks associated with both short- (acute) and long-term (chronic) exposures [3]. For acute risk, the EEC may be determined by an upper limit of daily averages predicted from an exposure model and divided by an acute LC/EC_50_ value to obtain an RQ. In chronic risk estimation, the EEC is based on a threshold of longer exposure duration (e.g., 60-days for fish; 21-days for aquatic invertebrates) and compared to an estimated chronic effect threshold, typically a no-observed adverse effect concentration (NOAEC). The RQs are compared to the LOCs to evaluate risk. For the sake of screening-level assessments, acute RQs may be helpful to inform which chemicals and exposure scenarios require further characterization, and we do not dispute their use in this context. However, for higher-tier (more refined) assessments conducted on chemical applications that have the potential to cause significant ecological impacts over longer durations, more sophisticated approaches than RQs are available to characterize risk. In this section, we discuss the current process of applying RQs to characterize risk of long-term exposures in higher tiered assessments and demonstrate how this one-size-fits all approach (1) can be confounded by oversimplification of both exposure and effects, (2) can lead to the mistaken perception that the magnitude of the RQ reflects the magnitude of risk, and (3) relies on safety factors, which have long been criticized for their arbitrary and unquantifiable uncertainty [15].

### 2.1. Estimating Exposure Concentrations

**Figure 1.**
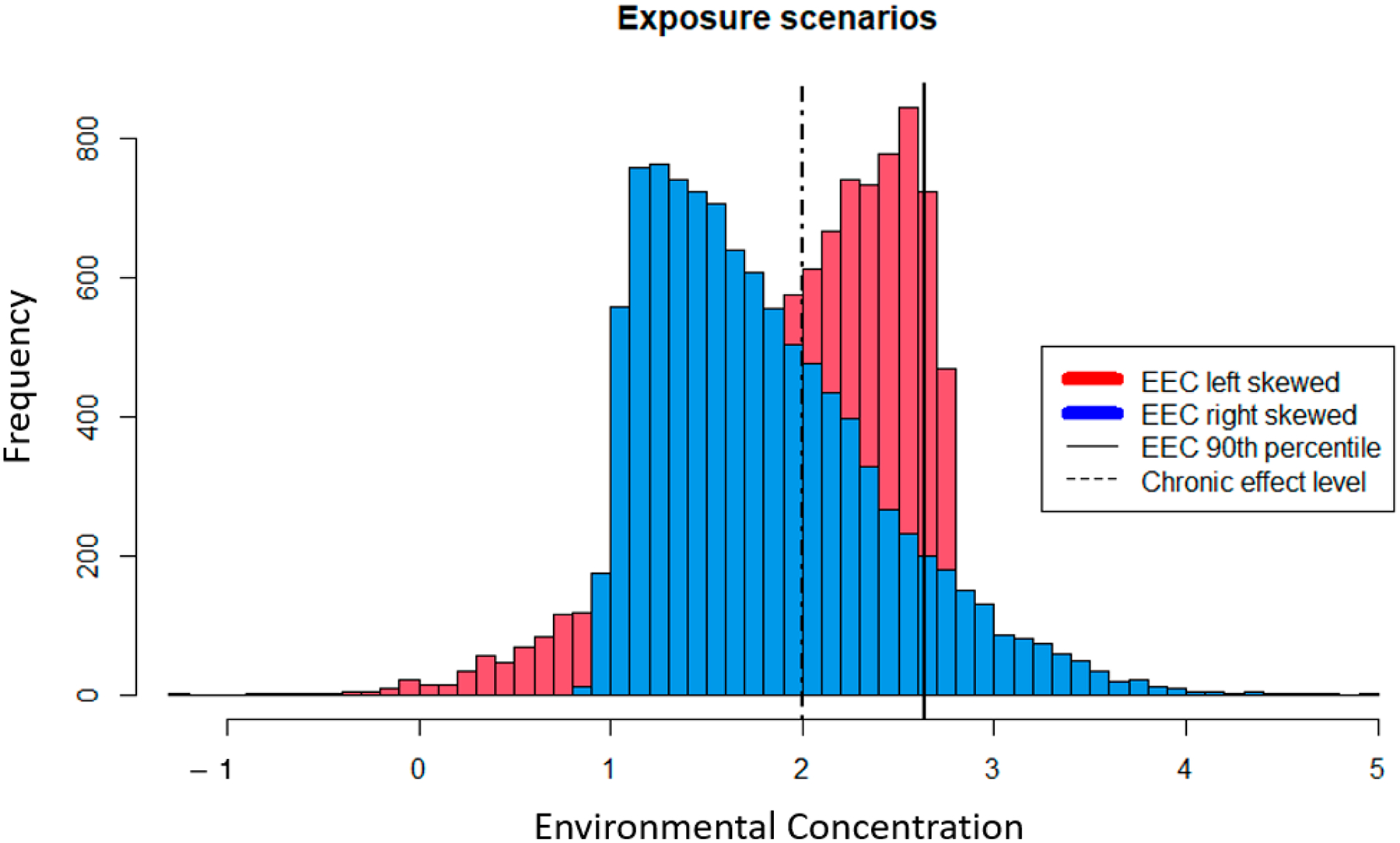
Hypothetical exposure profiles showing the frequency of chemical concentrations under different scenarios. Both distributions share the same 90th percentile noted by the solid line. A hypothetical chronic effect level is denoted by the dashed line for relative comparison of variable exposure profiles against a deterministic effect concentration.

While the methods used to calculate EEC point estimates are intended to be conservative, they do not utilize all available data to characterize exposure. For example, the USEPA pesticide risk assessment uses the 90th percentile of maximum annual 21-day average pesticide EEC as the numerator of the RQ for aquatic receptors [16]. However, the probability and magnitude of an adverse effect are driven by dynamic exposure profiles that cannot be readily represented by a deterministic EEC point estimate. Figure 1 demonstrates two chiral exposure distributions that differ in the directionality of their skew. In this hypothetical example, the 90th percentile of both distributions is 2.65, which we can imagine as some unit of concentration representing a 21-day average. If we assume a chronic effect threshold of two, both distributions will yield an RQ = 1.33. However, the frequency of EEC > 2 is greater for the left-skewed distribution (i.e., it is 0.33 and 0.57 for the right- and left-skewed distributions, respectively), whereas the right-skewed distribution contains concentrations that are two orders of magnitude greater than the highest concentrations realized by the left-skewed distribution. While these higher concentrations do not occur at a high frequency, the magnitude of toxic effect is concentration-dependent and ignoring the full scope of exposure profiles may have profound impacts on the risk assessor’s ability to adequately characterize risk. This example demonstrates how underlying variability in EEC data are not employed when using a scalar RQ given a constant effects threshold, and that point estimates of EECs may provide a false sense of conservativeness. While some risk assessors may employ variations of this approach on a case-by-case basis, the standard guidance continues to apply point estimates as described here.

Other critical elements of an exposure profile that are not captured by the deterministic EEC are the temporal and spatial distribution of concentrations. If the highest concentrations depicted by the right-skewed distribution in Figure 1 are present in the environment during a critical point in a species life cycle, an infrequent yet high spike in concentrations could have significant impacts on ecological receptors. Similarly, if the upper 10th percentile of EECs occurs in a location that is critical to species reproduction (e.g., nurseries), then risk will be missed using the 90th percentile value. Deterministic EECs are incapable of describing how the spatial and temporal heterogeneity of chemical and species distributions interact to inform the co-occurrence of chemical and receptor. Using the entire distribution of model-generated EECs to inform the likelihood and magnitude of an adverse effect does not require a significant increase in resources (i.e., time and data) or an overhaul of exposure modeling protocols, it just requires approaching the question with a larger, more comprehensive picture in mind and applying basic fundamentals of probability and statistics.

### 2.2. Estimating Effect Concentrations

As with EECs, effect thresholds strive to be conservative while omitting available information and relationships that inform actual risk. In general, the most sensitive apical (e.g., survival, growth, and reproduction) or subapical endpoint (i.e., can be directly and quantitatively linked to an apical endpoint) for the most sensitive species is used to derive a chronic effect threshold. The effect data are simplified as the hypothesis-based No Observed Adverse Effect Concentration (NOAEC), the Lowest Observed Adverse Effect Concentration (LOAEC), or the regression-based concentration at which x% effect is observed (e.g., EC_10_ and EC_20_). Such effects are measured at the individual level for surrogate species and these effects are neither linearly related to impacts on populations nor consistently translatable to populations of other species. While interspecies variation in sensitivity can span several orders of magnitude [17], if a chemical inhibits growth through some mechanism in one species of fish, it is assumed likely to inhibit growth in other species of fish via a similar pathway. While some variability in species sensitivity is expected based on morphological distinctions (e.g., body size) [18], the chemical mode of action can provide great insight into the potential effects that guide the utility of surrogate species with confidence. However, no effect measured on individuals of any species (apical or subapical) can truly inform risk without putting those effects in the context of life history, which varies considerably across closely related species and drives the actual outcome of chemical exposure on ecological receptors.

The ERA community has been distracted with discord over the use of the EC_x_ over NOAECs and LOAECs, e.g., [19,20]; however, when applied as the denominator of an RQ this debate is moot as the RQ method is inherently more flawed than any one of its components. Using chemical effects represented as NOAEC or EC_x_ values is rooted in the best available science of the 1980–1990s. The development of standard toxicity test protocols boomed in the 1980s, with the Organisation for Economic Co-operation and Development, the American Society for Testing and Materials, and the USEPA developing methods and protocols for reproducible, quality-controlled evaluations of toxic effects on organisms. Leading-edge research during this era focused on optimizing culture conditions, dosing systems, and identifying test species and measurement endpoints that could be applied and interpreted consistently by academic, private, and government laboratories globally and used to support regulatory decisions. Statistical considerations were incorporated into standard methods and initially relied on hypothesis testing to identify concentrations (e.g., NOAEC and LOAEC) at or above which statistically significant adverse effects were expected to occur. As early as the mid-1990s, researchers began expressing concern over the limitations and inappropriate interpretation of hypothesis testing used in the ERA process [21,22]. The 2000s saw increased advocacy for using the EC_20_ in place of NOAECs/LOAECs [23–25] without considering how either number was actually applied to characterize ecological- and not individual- risk. This critical flaw in effects assessment is consistent with that of using a point estimate for EECs where a single number is not capable of informing risk in a dynamic and diverse environment. Like exposure profiles, using the full set of available data quantitatively provides significantly more information about the likelihood and magnitude of toxic effects. In our view, the ecotoxicological community needs to embrace the variability and uncertainty of the data that are available, rather than continuing to debate which approach produces the superior point estimate.

**Figure 2.**
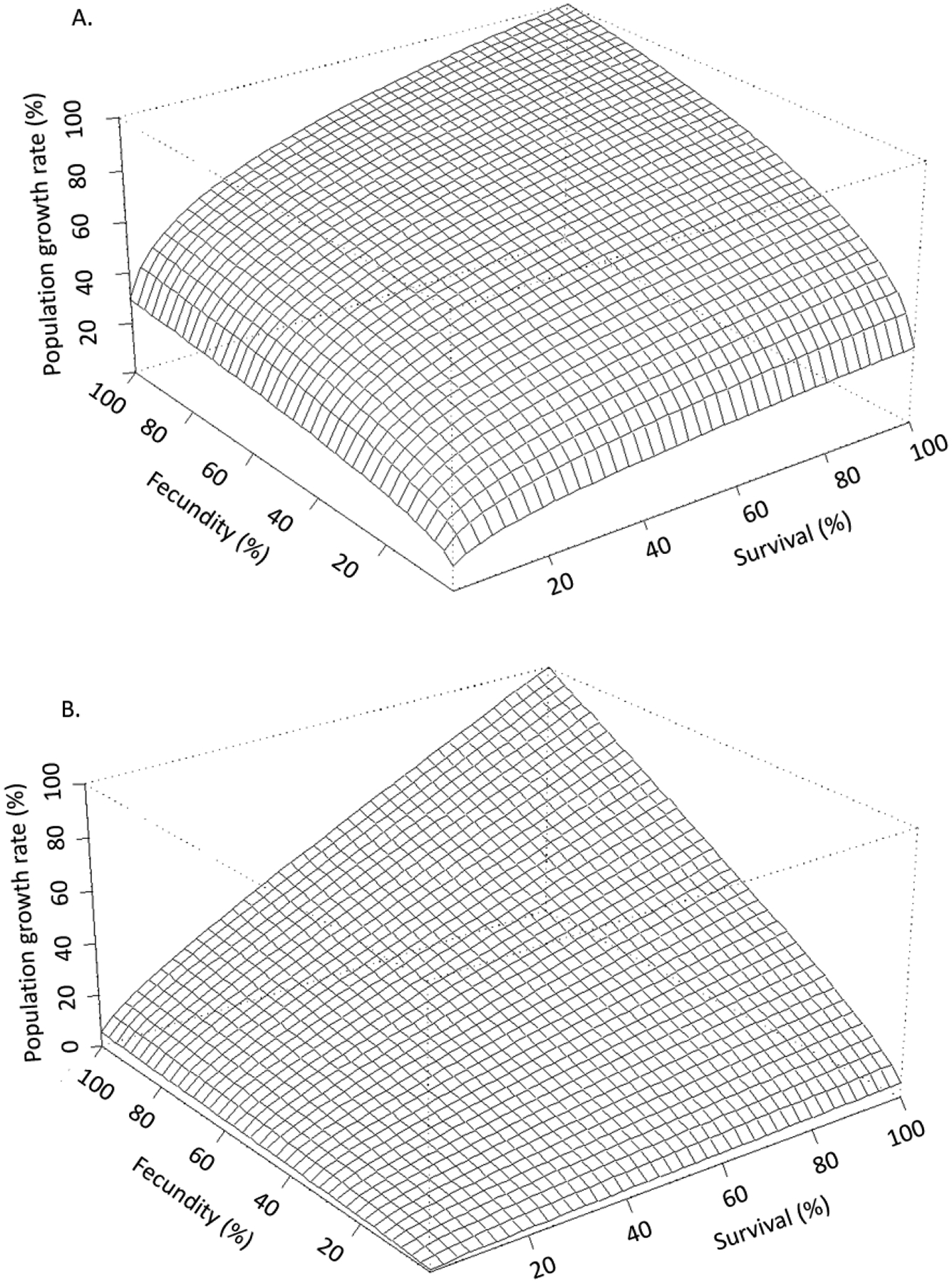
Stress response curves in 3-dimensions for hypothetical species exhibiting classical (**A**) “K-strategist” and (**B**) “r-strategist” life-history types (from Raimondo et al., [33], reprinted with permission).

**Figure 3.**
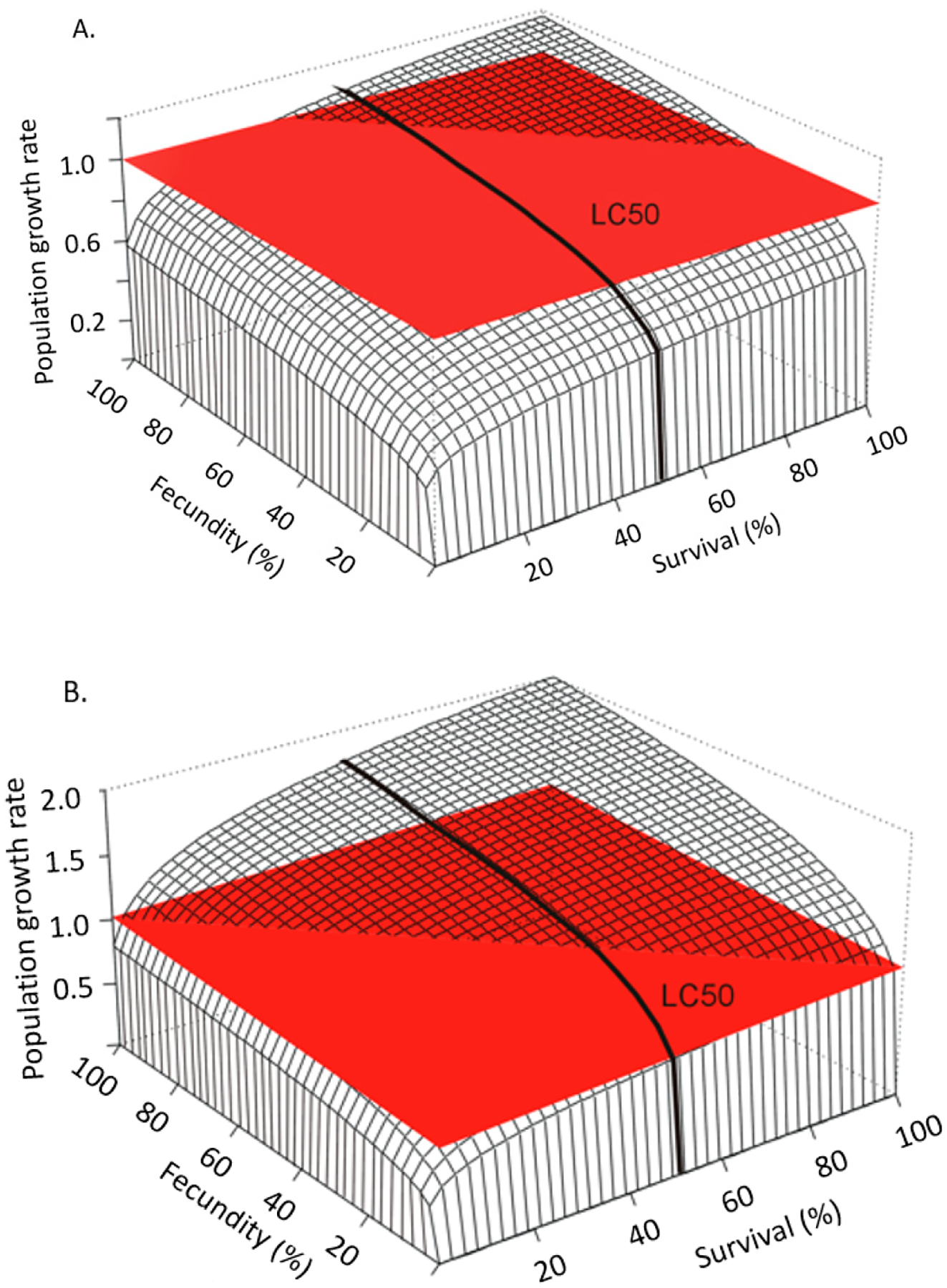
Demonstration of risk (i.e., λ < 1) as a function of life history and variability of risk tolerance around a point estimate (LC_50_) for (**A**) mysid shrimp and (**B**) gypsy moth with low and high population potential, respectively, as defined by the value of population growth rate (from Raimondo et al. [33], reprinted with permission).

For effects assessments to best reflect the available data they must be put in the context of life history [26–33]. Raimondo et al. [33] used simulations to demonstrate how populations of species with different life histories deterministically respond to proportional reductions in survival and reproduction caused by chemical stressors (Figure 2). In this example, such reductions would be informed by the whole dose-response measured during toxicity tests on a surrogate species. Proportional reductions are applied to a hypothetical “K-strategist”, for which reproduction by mature individuals is limited and the survival of the young is critical for population sustainability (Figure 2A), and a hypothetical “r-strategist”, for which females produce abundant offspring with a high natural mortality rate (Figure 2B). Figure 2 shows a stark contrast in the pattern of proportional reductions in population growth rate, *r*, (*z*-axis) with the same proportional reductions in survival (*x*-axis) and reproduction (*y*-axis). This is one simple example that shows how life history is the actual driver of how populations respond to stressors, a point that has been echoed extensively by population ecologists for decades [26,30,34], yet still not typically characterized in ERAs. In addition to the relative importance of survival and reproduction for any given species, risk is also influenced by a population’s potential to grow, which can be characterized by the magnitude of *r* or the finite growth rate, λ (*r* = ln(λ). A high population potential would be indicated by a high growth rate and is distinct from the relative contributions of survival, growth, and reproduction. In Figure 3, Raimondo et al. [33] used two species with the same patterns of λ decline with proportional reductions in survival and reproduction, but show that the two species differ in the magnitude of λ, as noted by the values of the z-axes. The red horizontal surface cuts through the 3-dimensional response curves at the point where the populations will decline (λ < 1), demonstrating that the species represented by Figure 3A is inherently less tolerant of chemical effects than the species represented by Figure 3B. The line denoted as the LC_50_ can be interpreted as an example point estimate measured on a surrogate species in the laboratory, which cuts across varying rates of population decline for the two species. While these deterministic simulations do not include environmental or demographic variability, they are useful to demonstrate that laboratory-based point estimates alone cannot fully inform risk. These examples are supported by numerous studies emphasizing that a percent effect (e.g., EC_x_) is not meaningful in and of itself [30,31,35].

### 2.3. Levels of Concern

Levels of concern essentially represent safety factors applied to RQs; however, it would be incorrect to assume that the magnitude of the RQ contains any real association with the magnitude of risk (Table 1). The USEPA guidance for developing ERAs acknowledges that RQs are not correlated with, or indicative of, magnitude or probability of risk [4], which is contrary to the application of LOCs to RQs. As demonstrated in Figure 1, two scenarios with the same RQ may differ in peak concentrations by orders of magnitude, and those concentrations can have a wide range of effects for species of different life histories (Figures 2 and 3). There is no ecological or biological logic to comparing RQs to LOCs to understand ecological risk and doing so implies a greater certainty in the relationship between RQ and risk than exists in reality. The National Research Council strongly recommended replacing safety factors with more robust, data-driven approaches to increase the relevance of ERAs, citing several alternative methods while echoing concerns voiced across the research community for decades [2,27,28].

At the heart of the RQ-LOC allure is a bright line amenable to decision making for a complex and dynamic environment. Indeed, a common criticism by risk managers of probabilistic methods, such as many population models, is that they lack a clear line with which to gauge whether a risk estimate exceeds a particular threshold. While bright lines may help facilitate the difficult role of risk managers, using bright lines to justify a scientific claim or conclusion can lead to erroneous interpretations [36]. As data used in, and interpreted for, ERAs are based on statistical testing (NOEC and LOEC) or model fitting (EC_x_), risk assessors and managers should adopt the best statistical practices. Recently, the American Statistical Association (ASA) published a statement discouraging the use of bright lines in decision making, highlighting the misuse and misinterpretation of *p*-values [37]. The ASA’s motivation to move the research community away from black or white “significance” and their reasoning is justly applicable to bright lines applied in risk management decisions. Wasserstein et al. [36] summarized the ASA statement into a cheat sheet of “don’t”s, which we extend to ERA-relevant language to advocate for the end of bright lines in the ERA process (Box 1). While it may seem as though “don’t”s encompass the entirety of the current effects characterization process, we discuss how the best available science to inform risk characterization already adheres to these recommendations in the next section, and offer recommendations for how risk assessors, managers, and researchers can move forward to implement such changes in closing.

## 3. The Need for Better, More Robust and Relevant Methods

### 3.1. Mismatch between What ERA Measures and What It Endeavors to Protect

In contrast to human health risk assessment, the ERA process is generally intended to protect populations and ecosystems and not individuals [3]. Even for threatened and endangered species, for which it might be argued that the loss of any individuals is to be avoided, risk assessments are intended to determine conditions under which species populations are likely to decline (i.e., to be in jeopardy). This implies that any measure of chemical effects used in ERAs need to either be a direct measure of population-level impact or linked quantitatively to such impacts. For some small and rapidly growing species (e.g., phytoplankton and rotifers), standard toxicity tests directly measure population growth rate. For most species of interest, however, it is impractical to experimentally measure chemical impacts on population growth directly, and most toxicity tests measure effects on individual survival, growth, or reproduction. As was demonstrated above, the relationship between individual-level effects of chemicals and their population-level impacts varies as a function of species life history as well as spatial and temporal aspects of the chemical exposure scenario. In addition, interactions among individuals in populations (e.g., density dependence), the presence of other environmental drivers, and stochasticity in all of the above mean that most toxicity test measurement endpoints are not likely to be useful proxies for population-level effects of chemicals.

Different levels of complexity are required for different ERAs depending on the nature of the risk being characterized [38]. Although population models can accommodate different levels of complexity, this flexibility has been a historical hindrance in developing and applying population models in ERAs [11]. The pushback from regulatory agencies has focused on the challenges and limitations of population models [4] without properly comparing model limitations with those of RQs. In these cases, arguments against using models are typically presented without comparison to existing approaches (i.e., RQs) and thus the weaknesses of current practices are not explicitly acknowledged or held up against alternative approaches.

With the continued advancement of technologies that facilitate the rapid and inexpensive detection of chemical effects at the biochemical and molecular levels, the need for approaches to link such measures to apical endpoints of regulatory interest that matter for ERAs (i.e., impacts on populations) are essential. The adverse outcome pathway (AOP) framework was introduced to conceptually link the effects of chemicals across levels of biological organization, incorporating mechanistic understanding, and in some cases a quantitative understanding, of how responses at lower levels could lead to effects at higher levels [39]. Current efforts are moving towards further enhancing AOPs from a conceptual, descriptive framework to a more quantitative, mathematical approach, e.g., [40]. Moving forwards with the use of AOPs as decision support tools for ERAs will require that they are incorporated into quantitative models that produce outputs of relevance for the process [41].

### 3.2. A Brief History of Population Modeling in the Context of ERA

Population modeling has a long history, usually traced back to Thomas Malthus’ 18th century study of human demography and his Malthusian growth model [42]. The earliest applications of population models for decision support are in fisheries, dating back to the Danish biologist, Carl Georg Johannes Petersen [43]. Although early models were relatively simple, and limited by both ecological theory and computational power, today’s models are substantially more sophisticated and remain as essential management tools in fisheries and wildlife management [44]. Today, population dynamics theory and models are essential components of every introductory ecology textbook as well as textbooks focused on describing the ERA process, e.g., [29]. Entire textbooks covering the details of specific types of models, such as matrix population models [45], metapopulation models [46], and agent-based models (ABMs) [47] are widely available and have been for some time. The argument that the science underlying the use of population models is not robust, verified, or otherwise appropriate for regulatory decision making is false. The foundation of ecological models is considerably older and better understood and accepted by the general science audience than the exposure models regularly used and applied as the basis of exposure characterization in ERAs.

The discussion of population models as tools for conducting ERA dates at least as far back as the 1980s, e.g., [48,49]. Since that time, several books, e.g., [29,50], and numerous scientific review articles [51] have been published on this topic. A number of international workshops sponsored by the Society of Environmental Toxicology and Chemistry have been held, including in Roskilde, Denmark, in 2004 [50], Leipzig, Germany, in 2007 [32], Le Croisic, France, in 2012, and Monschau, Germany, in 2013 [52]. A USEPA Risk Assessment Forum Technical Workshop on Population-Level Ecological Risk Assessment was held in Washington (DC, USA) in 2008 to develop guidance for the USEPA programs [53]. The guidance on good modeling practice for ERAs received a major boost in 2009 with the EU-funded Marie Curie Training Network “CREAM”, (Chemical Risk Effects Assessment Models) in which both specific models and general guidance for good modeling practice were developed [54]. Case studies were developed by a tripartite group of academic, industry, and regulatory scientists during the MODELINK series of workshops to demonstrate how population models could be applied to specific regulatory questions related to pesticide ERAs [52].

Efforts by the USEPA, the US Fish and Wildlife Service (USFWS), and the National Marine Fisheries Service (NMFS) to reach a consensus on approaches for assessing risks to federally threatened and endangered (i.e., listed) species from pesticides led to the publication in 2013 of an influential report by the US National Academy of Sciences [28]. This report defined a specific three-tier assessment process in which the USEPA performs the screening level and intermediate ERAs while the USFWS or the NMFS conducts the third-tier ERA. Under this framework, population models can be used to translate laboratory data collected on surrogate species to ecologically relevant endpoints for listed species and could be used to answer different questions in the second and third tiered assessments. Ultimately, the USFWS and the NMFS could apply such models to evaluate whether the chemical exposure could result in jeopardy to the listed species. This renewed interest in population modeling for ERAs led to further attention in the form of industry sponsored (CropLife America) workshops and focused sessions at major conferences (SETAC, American Chemical Society). However, to date, limited progress has been made in applying population models to these assessments, as the methods used for model parameterization remain contentious. Recent biological evaluations (BEs) that have been published thus far under the new process rely entirely on individual-level effect endpoints [55–57], perpetuating the use of the NOAEC and the ECx. Meanwhile, Europe continues to move forwards alongside the science, slowly but surely, providing guidance for good modeling practice for mechanistic effect models used for pesticide ERAs [58] and working constructively with modelers and industry to refine specific models (e.g., BEEHAVE) [59], such that they could be incorporated in the ERA toolbox.

### 3.3. Current State of the Science to Support ERA

The science available for conducting ERAs has advanced considerably since the late 1990s when the USEPA’s general ERA guidance was last updated. While the USEPA’s ERA process under some regulatory statutes continues to evolve (e.g., >700 guidance documents for ERA conducted under FIFRA), these updates and additional guidance are not fully captured in agency-wide guidance, and the RQ-LOC continues to be the predominate endpoint for chronic risk characterization. Meanwhile, a variety of mechanistic effect models are available to allow the quantitative and mechanistic extrapolation of organismal or suborganismal responses to chemicals measured in most toxicity tests to endpoints that matter for ERAs (i.e., population-level responses). Likewise, toxicokinetic–toxicodynamic (TK–TD) models allow chemical uptake and effects to be estimated from the kind of temporally varying chemical exposures that often characterize field conditions. Models such as MCnest [60] make simple use of life-history information for a diversity of bird species to translate toxicity test endpoints into metrics relevant to ecological considerations, and similar so-called translator models are in various stages of development for fish [61], amphibians [62], and invertebrates [63]. When put into a landscape context, spatial variability in chemical exposure can also be integrated with such models to assess how exposing different proportions of a population to a chemical may influence risk.

Accolla et al. [13] reviewed the key features of population models of relevance for use in the ERA process. They categorized models into three main types: unstructured, structured (e.g., matrix models), and agent-based. Unstructured models are simple, scalar models that can be as simple as *r* = births/deaths. Structured models represent populations broken down by relevant life stages, sizes, ages, or other distinctions that are relevant to the species. For example, structured models for amphibians could distinguish between aquatic larval stages and terrestrial adult stages, as the different life stages are exposed to chemicals differently based on their habitat. Agent-based models (ABMs) consider distinctions among autonomous units, such as individuals or cohorts that are more refined than age groups or life stages. Any of these types of models can incorporate density dependence, spatial heterogeneity, external drivers, stochasticity, life-history traits, behavior, energetics, and the integration of exposure and effects. Whether or not to include these components in the model structure depends on the ERA context, how each feature could be incorporated into the different model types, and the available data to inform how they function within the model. In their simplest form, population models can integrate multiple individual-level toxicity test endpoints into a measure of population growth rate, e.g., [26,31,64], while excluding many environmental features that are either unknown or not applicable at a large-scale, such as national levels. Etterson and Ankley [65] use a very simple demographic model to reiterate the importance of incorporating life history with toxicological effects in a manner similar to that demonstrated here in Figures 2 and 3. When used in even their simplest form, population models can compare the relative vulnerability of different species to impacts on their life-history traits as well as their inherent potential for recovery, all else equal. In their most complex form, population models can project landscape-level changes in populations or species through space and time by overlaying exposure profiles, landscape attributes, and population dynamics, e.g., [9]. These models easily lend themselves to incorporating uncertainty or variability of all influential parameters to derive probabilities of various impacts and how mitigation efforts may alter such outcomes. However, limitations of resources and data may constrain the complexity with which population models can be developed for a particular ERA. But as demonstrated by Raimondo et al. [11], not all ERA objectives require complicated models, and models are still valuable in estimating the likelihood of adverse effects when developed using limited data scenarios and incorporating the best available quantitation of variability.

With more features included for increased complexity, population models can be very effective at distinguishing adverse changes due to chemical exposure from those within the normal pattern of ecological variability or associated with other stressors (e.g., climate change and habitat loss). Natural fluctuations in environmental conditions, intra- and interannual variability in population size and structure, and cyclic events of various periods (e.g., bird migration and tides) are very important in natural systems, may mask or delay stressor-related effects, and are important considerations in ERAs [4]. The RQ-LOC bright line approach is incapable of accounting for these factors. Another important advantage of population models over RQs for assessing chronic chemical risk is that they can use all of the available data on exposure and effects (i.e., the entire dose–response curve, rather than a point estimate such as EC*x*). Unlike RQs, population models are designed for quantitative uncertainty and sensitivity analyses, which will help decision makers gauge the confidence with which their assessments are associated. Moreover, population models are especially good at evaluating the adverse changes in assessment endpoints, the nature and intensity of effects, the spatial and temporal scale, and the potential for recovery and mitigation.

Population models allow risk assessors and managers to consider “what if” scenarios and to forecast beyond the limits of observed data and techniques that are based solely on empirical data [4], which is exactly how risk assessors have used exposure models for decades. Not only are population models well suited to quantifying the likelihood of impacts of chemicals on populations, they are also ideal for modeling a population’s recovery following an adverse event such as chemical exposure or in response to different management actions. For example, Vaugeois et al. [66] developed an ABM to quantify the effects of chemicals impairing different energetic pathways related to feeding, reproduction, growth, or maintenance in lake sturgeon (*Acipenser fulvescens*). They explored how such impairments of energetic pathways, through their effects on sturgeon life-history traits, impacted population abundance and recovery in the absence of exposure. Furthermore, they simulated different management strategies to aid sturgeon population recovery. Because these fish live for around 150 years and do not start reproducing until they are approximately 20 years old, it can take a very long time to determine whether management efforts are having the desired effect. Comparing different management strategies in computer simulations of 100 years or more can greatly improve the cost-effectiveness of population recovery initiatives and the protection of long-lived species [67].

In addition to the scientific advances in population modeling over the last several decades, there have been vast improvements in the development of guidance related to issues such as appropriate level and type of complexity that needs to be incorporated, how specific or general the models need to be, and the extent to which interactions through competition and trophic relationships need to be incorporated [14,15]. As described in the Introduction, much effort by the scientific community has focused on developing guidance for both model developers as well as those in a position to use models for decision support. Thus, a lack of guidance can no longer be justified as a reason for not using these types of models in ERAs [11].

While population models have extensive flexibility and power to inform responses beyond a single point estimate such as a NOEC or ECx, they contain limitations and uncertainties inherent in all models. More complex models require more information, and such data may not be available or may need to be simulated using the qualitative understanding of relationships. For example, density dependence may be an important component of a population to self-regulate, but it may be very difficult to obtain quantitative functions for how population density influences growth rate. Models can range in their spatial resolution from simple life-history models that do not consider landscape features, to spatially explicit representations of a particular landscape. However, as Pop-GUIDE demonstrates through the papers of this special issue, not all assessments require complex models or complex features. Simple density-independent life-history models (e.g., Pop-GUIDE’s “general” model) may not be precise in their predictions of population density but are able to inform changes in a population’s potential or “fitness” (Figures 2 and 3) [65]. Model developers and risk assessors should be clear on what models are capable of informing and communicate that transparently. Additionally, the variability and uncertainty of models can also be difficult to evaluate, and users may only be able to validate functions within the model (i.e., concentration–response functions). Transparency of the confidence or uncertainty of data and functions used in the models should be included in all model documentation [8–14].

## 4. Take Home Messages

The research supporting the use of population models (or mechanistic, demographic models) as the best available science for ERAs is extensive, comprehensive, and exhaustive [8–15,26–35,41–47,50–54,60–66]. Meanwhile, the continued use of deterministic RQs-LOCs is side-tracked by discourse over the interpretation and relevance of NOAECs and ECxs. We contend that it is long past time to further advance chronic risk characterization by making greater use of population models to refine assessments and inform regulatory decisions. Perhaps the biggest reason for pushback from risk assessors and risk managers on using such models in ERAs is that the models do not tell them which impacts are acceptable and which are not. This is not a flaw of the models but does highlight that decisions about acceptability do have to be made. At some time in the past, such decisions were made with respect to LOCs and RQs, and so no additional effort is needed for risk managers today to “decide” whether an acute RQ > 0.5 for an aquatic species indicates high risk. Now that the science has advanced to provide more ecologically relevant and nuanced measures of risk based on population (and other mechanistic effect) models, what is needed now is to identify a standard suite of models to use for different types of ERAs as well as transparent and consistent criteria for deciding which risks are acceptable and under what conditions.

The papers featured in this Special Issue of *Ecologies* demonstrate how population models developed according to the latest guidance (i.e., Pop-GUIDE), provide a more scientifically robust basis for conducting ERAs that incorporates key aspects of species life history and ecology, make efficient use of the available data, and produce outputs that are better aligned with things people care about. Etterson [68] used four bird models with different degrees of realism to demonstrate the important role that population models can play at different tiers of the ERA process. Garber et al. [69] developed and applied a simulation model of honeybee (*Apis mellifera*) colonies. *A. mellifera* is a common test species used in pesticide ERAs, and is therefore relatively data-rich, which facilitates model parameterization and evaluation. For many species, however, data may be difficult or impossible to collect. In such cases, population models can make effective use of the data that are available and, while perhaps not providing precise measures of risk, can produce robust estimates of relative risk under various scenarios. Three of the papers focus on how population models can improve ERAs for threatened and endangered species. Accolla et al. [70] used an ABM to demonstrate how differences in life history among four species of Cyprinid fish, together with species–specific differences in density dependence, influence their responses to realistic fungicide exposure. Awkerman and Greenberg [71] used a matrix population model to show the importance of altered hydroregime, in combination with pesticide exposure, on the population dynamics of the threatened southern toad, *Anaxyrus terrestris*. Miller et al. [63] showed the value of combining a population model with a field assessment and laboratory-based chemical analysis to assess the risks of an organophosphorus pesticide to the threatened vernal pool fairy shrimp, *Branchinecta lynchi*. Examining a very different management challenge, Hudina et al. [72] developed a conceptual population model to identify possible management scenarios for the invasive signal crayfish, *Pacifastacus leniusculus* and expanded Pop-GUIDE to increase its applicability to other fields beyond ERA.

We summarize key take home messages from this perspective that are demonstrated throughout this special issue:

1. The environment is not deterministic, so we cannot rely solely on deterministic methods to understand its future;
2. Higher-tier risk assessments should utilize the entire domain of data to transparently explain decisions based on variability and uncertainty within all available information;
3. Incorporating species life histories as well as spatial and temporal considerations is critical to understanding risk;
4. Holding on to “bright lines” (e.g., RQ-LOCs) for their ease of use is not an acceptable justification for putting the environment at risk by using outdated methods;
5. Stakeholders will need to come to a consensus on how to interpret different ways of expressing risk that does not require risk managers to have to re-evaluate acceptability for every new ERA;
6. The general USEPA ERA guidance [4] does not accommodate advances in the state of the science, therefore an update of this guidance is warranted.

Although the role of RQs-LOCs may remain useful for simple, screening-level assessments based on simple metrics at the lowest tiers, ecological risk assessors and managers should start envisioning ERAs in a post–bright line world for higher tiered assessments evaluating chronic risk. In doing so, it will be important to design an approach that can be applied transparently, consistently, and equitably across different types of ERAs. There may be cases where ecological risks cannot be mitigated but are deemed “acceptable” and “reasonable” if the benefits (e.g., economic, health, social, and political) outweigh the risks. However, when risk is properly characterized, the assessment is easier to explain, justify, and defend [38]. Comprehensive model guidance, such as Pop-GUIDE, provides a means for making greater use of the available data towards characterizing potential risks. As the papers in this special issue show, the application of Pop-GUIDE as a tool for model development and documentation is ready for application in ERAs under various regulatory statutes.

## Figures and Tables

**Table 1.** Levels of Concern (LOC) used in USEPA pesticide risk assessments [5].

Exposure Duration	Taxa	LOC (RQ > Limit)	Interpretation
Acute	Aquatics	0.5	High acute risk
		0.1	Risks may be mitigated through restricted use
		0.05	Eendangered species may be affected acutely
	Mammals and birds	0.5	High acute risk
		0.2	Risks may be mitigated through restricted use
		0.1	Endangered species may be affected acutely
	Bees	0.4	Acute risk
Chronic	All taxa	1	Presumption of chronic risk
	Endangered species	1	May be affected chronically

## Box 1. Guidance from the ASA [36]:

1. ASA: Do not base your conclusions solely on whether an association or effect was found to be “statistically significant” (i.e., the *p*-value passed some arbitrary threshold such as *p* < 0.05).
  ERA: Do not base your conclusions solely on whether a single threshold is exceeded.
2. ASA: Do not believe that an association or effect exists just because it was statistically significant.
  ERA: Statistical significance does not consistently translate into biological or ecological significance.
3. ASA: Do not believe that an association or effect is absent just because it was not statistically significant.
  ERA: Do not ignore variability that is around summary values.
4. ASA: Do not believe that your *p*-value gives the probability that chance alone produced the observed association or effect or the probability that your test hypothesis is true.
  ERA: Do not believe that RQs and LOCs reflect the probability of adverse outcomes
5. ASA: Do not conclude anything about scientific or practical importance based on statistical significance (or lack thereof).
  ERA: Same
